# Histopathological and Clinical Findings of Biopsy-Proven Focal and Segmental Glomerulosclerosis: A Retrospective Study

**DOI:** 10.7759/cureus.23083

**Published:** 2022-03-11

**Authors:** Hanadi M Alhozali, Renad A Ahmed, Rasana B Albeirouti, Fahad A Alotibi, Deemah K Ghazi, Mohammad A Shikdar, Maha K Alghamdi, Reem A Al Zahrani

**Affiliations:** 1 Faculty of Medicine, Department of Medicine, Nephrology Unit, King Abdulaziz University Hospital, Jeddah, SAU; 2 Department of Medicine, King Abdulaziz University, Jeddah, SAU; 3 Internal Medicine, King Abdulaziz University, Jeddah, SAU; 4 Department of Pathology, King Abdulaziz University, Jeddah, SAU

**Keywords:** outcomes, histopathology, biopsy, focal segmental glomerulosclerosis, fsgs

## Abstract

Background

Focal segmental glomerulosclerosis (FSGS) is characterized by the presence of glomerular damage on histopathological examination. The major defining symptom of FSGS is proteinuria, which indicates damage to the glomerular filtration barrier. Additionally, FSGS is the most common cause of primary nephrotic syndrome. However, in Saudi Arabia, there is a paucity of research on this topic. Therefore, this study was designed to examine the clinical features, laboratory findings, and presence of comorbidities in patients with FSGS to determine their effects on clinical outcomes.

Methods

We retrospectively analyzed the histopathological and clinical data of patients diagnosed with FSGS via biopsy at King Abdulaziz University Hospital, Jeddah, Saudi Arabia, during the period 1989-2020. Biopsy samples were labeled according to the Columbia classification as tip, perihilar, cellular, collapsing, or not otherwise specified (NOS).

Results

We included 39 children and 21 adults. Males accounted for 54.1% of the sample. Hypertension was the most common comorbidity. Regarding FSGS subtypes, 60.9% of the lesions in the adult patients were collapsing lesions, followed by NOS (26.1%). In pediatric patients, 36.8% of the lesions were NOS, followed by collapsing lesions (28.9%). We also observed a very low rate of remission. In both age groups, the most common clinical presentation was nephrotic syndrome.

Conclusion

We found a high prevalence of collapsing and NOS FSGS subtypes in both the adult and pediatric age groups. The most prevalent outcome was the persistence of nephrotic syndrome with low rates of remission.

## Introduction

Focal segmental glomerulosclerosis (FSGS) is a morphological kidney injury that is histologically characterized by focal and segmental fibrosis of glomeruli [1]. FSGS is typically diagnosed from histological findings of renal tissue biopsies combined with laboratory findings [2]. The slit diaphragm, which prevents protein leakage into the urine space, is formed by specific tight junctions between podocyte foot processes in a typical glomerular structure [3]. Notably, a previous study that performed electron imaging on biopsies from FSGS patients observed deformations of the typical architecture (or effacement) of podocyte foot processes [4]. The clinical findings of FSGS include proteinuria, hematuria, hypertension, renal insufficiency, and nephrotic syndrome (NS), leading to end-stage renal disease (ESRD), which has a high morbidity and mortality rate [4].

Furthermore, FSGS has been found to be the underlying cause in approximately 55% of adults presenting with NS [5]. A study conducted in Asia found that patients with FSGS had the highest incidence of ESRD and the fastest decline in their glomerular filtration rate (GFR) [6]. Additionally, in the last two decades, data collected by the United States Renal Data System and the National Center for Health Statistics revealed that the annual incidence of ESRD in FSGS patients had grown 11-fold and occurred twice as often in males than in females [7]. 

Although studies of renal specimens from different US institutions have found that the incidence of FSGS with complex unclear causes of ESRD has increased significantly, they suggest that the increase in ESRD is most likely due to the rise of FSGS cases reported in the past 10-20 years [8]. The impact of FSGS can further be seen locally; for example, a study conducted in the Kingdom of Saudi Arabia revealed that the most common type of primary glomerular disorder observed in clinical practice is FSGS [9]. Several morphologic variants of FSGS have been described, with the frequency of the variant depending on the demographic characteristics of the population studied. According to the Glomerular Disease Collaborative Network and the Columbia University Group, the frequency of FSGS was as follows: tip (37%), not otherwise specified (NOS; 32%), perihilar (26%), and collapsing (5%) [10]. A study that included a pediatric sample with a high percentage of African Americans found that NOS was the most common variant (44%), followed by cellular (32%) and collapsing (24%) variants. Notably, neither tip nor perihilar lesions were represented in their sample [11]. FSGS is classified as primary, genetic, or secondary based on the latest Kidney Disease Improving Global Outcomes guideline [12], with multiple risk factors for developing the disease that are yet to be discovered [13].

Therefore, a better understanding of the disease pattern and prevalence of FSGS as well as the development of effective therapeutic options for glomerular protection would significantly impact public health. To the best of our knowledge, no study has been conducted in the western region of Saudi Arabia to assess whether FSGS subtypes are associated with disease outcomes. Therefore, the aim of this study was to determine the common histological variants of FSGS, clinical presentation and their clinical outcomes in pediatric and adult patients.

## Materials and methods

This retrospective study was conducted at King Abdulaziz University Hospital (KAUH), a teaching hospital in the western region of Saudi Arabia. Patients were followed up from 1989-2020 at the Division of Nephrology, Department of Medicine of KAUH. This study was approved by the ethics committee of KAUH (reference number, 174-21). Patients’ data were obtained through the hospital’s electronic records, which were later collected via electronic data collection sheets and transferred into Excel sheets for interpretation.

Patients and clinical data

Patients were defined as pediatric if they were < 18 years of age and adults if ≥ 18 years old. We included all hospitalized patients who were referred to the department of nephrology and were diagnosed with FSGS based on clinical assessments, laboratory findings, and biopsy results. However, patients with minimal change disease were excluded from this study. Patients’ demographic data included medical record number, age at diagnosis, gender, nationality, height, weight, body mass index (BMI), and blood pressure.

Sixty of the 350 renal biopsy samples analyzed for FSGS were collected. According to the Columbia classification, each FSGS biopsy was further investigated and classified into one of the five categories of FSGS (tip, perihilar, cellular, collapsing, or NOS) based on the results obtained from light microscopy, immunofluorescence, and electron microscopy [4]. Levels of serum albumin, total cholesterol, low-density lipoprotein, hematuria, serum creatinine, and proteinuria were noted. All laboratory tests were performed at the following time points: at the time of biopsy, after three months, after six months, after one year, after two years, and finally at the time of the last follow-up. The results obtained were used in the calculation of glomerular filtration rate (GFR) using the Chronic Kidney Disease Epidemiology Collaboration (CKD-EPI) creatinine equation (2009) for adults and the creatinine based on the “bedside Schwartz” equation (2009) for pediatric patients at the time points mentioned above. 

Furthermore, we reviewed the medications administered to these patients to gain insight into how the hospital approached treatment of the disease. These medications included glucocorticoids, cyclosporine, angiotensin-converting enzyme inhibitors, and lipid-lowering agents. Moreover, we collected information regarding any associated comorbidities, including diabetes mellitus, hypertension, ischemic heart disease, chronic heart failure, malignancy, cerebrovascular disease, autoimmune disease, thyroid disease, and peripheral and vascular diseases.

Definitions

We classified the outcome of these patients into four categories: chronic kidney disease (CKD), ESRD, remission, and no remission. ESRD was defined as estimated glomerular filtration rate (eGFR) ≤ 15 mL/min or the need for renal replacement therapy. A partial remission was considered if proteinuria was < 2.0-3.0 g/24h but > 0.3-0.5 g/24h [14]. However, CKD was interpreted in adults based on the estimated GFR (<60 mL/min/1.73 m2) or if they clinically presented with CKD [14]. No remission was characterized by proteinuria ≥ 3.5 g/24h [15]. We identified hypertensive patients with a systolic blood pressure ≥ 130 mmHg and/or diastolic blood pressure ≥ 80 mmHg, according to the American Heart Association’s report [16].

Statistical analysis

Data analysis was performed using the Statistical Package for Social Sciences (SPSS) version 21 (IBM Corp., Armonk, NY, USA). Qualitative data were presented as frequencies and percentages. Independent t-tests were performed to compare continuous variables between groups, and the results were presented as measures of central tendency (mean ± standard deviation and median). The Chi-square test was used to compare between categorical variables. Statistical significance was defined at p < 0.05. 

## Results

Of 60 cases with biopsy-proven FSGS, 39 were pediatric and 21 were adult patients. The mean age of the pediatric patients was 7.13 ± 5.18 years, while that of the adult patients was 35.82 ± 14.32 years (Table 1). Males were more represented in both age groups (55%). Among pediatric patients, the most common comorbidities included hypertension (HTN) (23.7%), diabetes (7.9%), and dyslipidemia (5.3%), while in adult patients, HTN (50%) and dyslipidemia (9.1%) were most common. A list of other comorbidities and their frequencies are presented in Table 2. 

**Table 1:** Demographic, clinical, and laboratory data Abbreviations: Cr; Creatinine, eGFR, estimated glomerular filtration rate; LDL, low-density lipoprotein; SD, standard deviation.

Variables	Adults		Children	
	Mean	SD	Mean	SD
Age (years)	35.82	14.32	7.13	5.18
Body mass index (kg/m^2^)	21.14	8.23	-	-
Systolic pressure (mmHg)	138.67	16.01	116.69	21.91
Diastolic pressure (mmHg)	85.33	16.29	74.31	17.45
Serum Cr (µmol/L)	131.45	74.66	43.36	53.06
Proteinuria (g/dL)	8.29	10.27	15.47	19.45
Cholesterol (mmol/L)	6.22	2.50	14.28	17.29
LDL (mmol/L)	4.33	1.39	5.84	2.46
Serum albumin (g/dL)	25.72	9.76	22.21	12.16
Proteinuria after 3-month (g/dL)	2.15	1.81	0.95	1.03
Proteinuria after 6-month (g/dL)	2.36	3.17	0.69	0.82
Proteinuria after 12-month (g/dL)	2.02	2.89	0.63	0.069
Proteinuria after 2-year (g/dL)	1.42	2.51	0.54	0.73
Serum Cr after 3-month (µmol/L)	117.79	83.60	65.29	116.28
Serum Cr after 6-month (µmol/L)	115.07	67.52	86.55	132.97
Serum Cr after 12-month (µmol/L)	179.49	181.10	89.73	150.36
Serum Cr after 2-year (µmol/L)	126.79	76.46	148.97	289.77
eGFR after 3-month (mL/min)	79.45	45.03	165	104.61
eGFR after 6-month (mL/min)	76.93	38.65	135.07	86.88
eGFR after 12-month (mL/min)	70.00	44.04	142.77	88.80
eGFR after 2-year (mL/min)	70.00	35.20	129.10	73.87

**Table 2:** Comorbidities among the two groups Data are presented as frequency (percent).

Comorbidities	Children	Adults
Hypertension	9 (23.7)	18 (85.71)
Diabetes mellitus	3 (7.9)	1 (4.5)
Dyslipidemia	2 (5.3)	2 (9.1)
Chronic heart failure	1 (2.6)	0 (0.0)
Peripheral vascular disease	1 (2.6)	0 (0.0)
Acute kidney injury	0 (0.0)	1 (4.5)
Ischemic heart disease	0 (0.0)	0 (0.0)
Cerebrovascular disease	0 (0.0)	0 (0.0)
Malignancy	0 (0.0)	0 (0.0)
Autoimmune disease	0 (0.0)	0 (0.0)
Sickle cell anemia	0 (0.0)	0 (0.0)
Thyroid disease	0 (0.0)	1 (4.5)

Renal biopsies revealed that segmental lesions were more common than global lesions in all age groups; however, the FSGS subtypes differed between the pediatric and adult patients. The subtype with the highest frequency in the pediatric group was NOS 39.5%, followed by collapsing FGGS 31.6%. Conversely, adults showed an opposite pattern, with collapsing 63.6% being the most frequent subtype, followed by NOS 27.3%. The frequencies of the other subtypes are shown in Table 3. 

**Table 3:** Histological findings, treatment, and final outcomes among the two groups Abbreviations: ESRD, end-stage renal disease; CKD, chronic kidney disease; NS, nephrotic syndrome. Data are presented as frequency (%).

	Children	Adults
Histopathological findings		
Collapsing	12 (31.6)	14 (63.6)
Cellular	7 (18.4)	1(4.5)
Tip lesion	0 (0.0)	0 (0.0)
Perihilar	4 (10.5)	1(4.5)
Not otherwise specified	15 (39.5)	6 (27.3)
Treatment		
Steroid	33 (86.8)	13 (59.1)
Angiotensin-converting enzyme	24 (63.2)	7 (31.8)
Cyclosporine	15 (39.5)	3 (13.6)
Lipid-lowering agent	2 (5.3)	5 (22.7)
Initial presentation		
NS	18 (85.7)	5 (71)
ESRD	0 (0.0)	0 (0.0)
CKD	3 (14.3)	5 (28.6)
Final outcome		
ESRD	5 (13.2)	1 (4.5)
CKD	5 (13.2)	5 (22.7)
Complete remission	4 (10.5)	5 (22.7)
Partial remission	1 (2.6)	3 (13.6)
No remission	33 (86.8)	14 (63.6)

Finally, mild tubular atrophy and mild interstitial fibrosis were more prominent than moderate and severe forms in both age groups (Table 4). At the time of biopsy, the following laboratory outcomes investigations were documented in pediatric patients: mean serum albumin, 22.21 ± 12.16 g/dL; serum cholesterol, 14.28 ± 17.29 mmol/L; proteinuria, 15.47 ± 19.45 g/dL; creatinine, 43.36 ± 53.06 µmol/L; and average GFR, 223.78 ± 183.88 mL/min/1.73 m2. In adult patients, the mean levels were as follows: serum albumin, 25.72 ± 9.7688 g/dL; serum cholesterol, 6.22 ± 2.50 mmol/L; proteinuria, 8.29 ± 10.27 g/dL; creatinine, 131.45 ± 74.66 µmol/L; and GFR, 68.90 ± 37.450 mL/min/1.73 m2. Hematuria was present in both groups, with a marked predominance in the 0-3 category (Table 5). 

**Table 4:** Renal biopsy results among the two groups Data are presented as frequency (%).

	Children			Adults		
	Mild	Moderate	Severe	Mild	Moderate	Severe
Tubular atrophy	9 (23.7)	2 (5.3)	0 (0.0)	8 (36.4)	5 (22.7)	0 (0.0)
Interstitial fibrosis	11 (28.9)	3 (7.9)	0 (0.0)	12 (54.5)	4 (18.2)	0 (0.0)
Arteriosclerosis	1 (2.6)	0 (0.0)	0 (0.0)	4 (18.2)	0 (0.0)	0 (0.0)
Arteriolar hyalinosis	0 (0.0)	0 (0.0)	0 (0.0)	2 (9.1)	0 (0.0)	0 (0.0)

**Table 5:** Presence of hematuria among the two groups Abbreviations: RBC, red blood cells; HPF, high-powered field.

Hematuria	Children	Adults
0–3 RBC/HPF	8 (21.1)	7 (31.8)
4–10 RBC/HPF	5 (13.2)	5 (22.7)
11–50 RBC/HPF	2 (5.3)	13 (4.5)
> 50 RBC/HPF	3 (7.9)	3 (13.6)

Regarding treatment options, the data were categorized based on the type of medication used to treat FSGS at KAUH. Glucocorticoid and angiotensin-converting enzyme inhibitors were administered at a significantly higher frequency than other medications in both age groups (Table 3). The most common presenting complication was NS in both age groups. In descending order, the final outcomes in both age groups were as follows: NS, CKD, ESRD, and eventually remission. The most common clinical presentation was NS in both age groups. We also found that males had a significantly higher GFR at the time of biopsy in the pediatric group (p < 0.005). Finally, no significant relationship was found between either hypertension or FSGS subtype with the disease outcome in either age group.

## Discussion

Low remission rate in relation to FSGS subtype

Our results show that the most common FSGS variant is collapsing, while the least common is the tip variant, with only a few cases of remission noted. In contrast, a study from Europe assessing FSGS variants in cases from 1980-2003 found that the most common variant was the tip variant, with higher remission rates observed among patients with this variant [10]. Based on the outcomes observed in our patients, we hypothesize that the low rate of remission in our study was due to a higher frequency of the collapsing variant compared to the tip subtype. In fact, previous studies have shown that tip lesions respond best to therapy and have promising results, unlike collapsing lesions [10-11]. Furthermore, a study by Thomas et al. revealed that the rate of remission in patients with the tip subtype was 53%, while in the collapsing subtype it was only 18% [15]. Finally, a study assessing the pathologic subtypes of FSGS also found that the tip subtype was associated with higher survival and remission rates than the collapsing subtype [4]. Based on these findings, it is likely that if more cases of the tip FSGS subtype were present in our study, the remission rates would have been higher.

Medication and outcomes

Regarding FSGS pathogenesis, it has been demonstrated that fibrin and platelet thrombi play a role in the early glomerular lesions associated with FSGS [13]. The presence of immunoglobulins (especially C3 and IgM) and fibrin in hyalinosis lesions suggests that the immune system is disrupted. Importantly, patients with corticosteroid-resistant FSGS may benefit from treatment with immunosuppressants, resulting in clearance of the accompanying NS [13,17]. In this study, however, the two most commonly prescribed medications were glucocorticoids and angiotensin-converting enzyme inhibitors.

Complete remission is a critical goal that predicts a good long-term prognosis, and because FSGS appears to be variable in terms of both the number of people who will remit and the time it takes for them to do so, there is a serious risk of over-treating the patient. According to a prior study, the length of treatment ranged from two to 50 months [18]. Importantly, it has been noted that if the upper limit is used, many patients will be treated for longer periods with a lower possibility of responding [18]. A study of 163 individuals with primary FSGS who received conventional first-line treatment, including daily steroids for four weeks, found that in the first round of treatment, 115 patients (71%) achieved complete remission, 23 patients (14%) had partial remission, and 25 (15%) had steroid resistance. In patients who achieved complete remission, the mean time to response (disappearance of proteinuria) was 2.7 ± 1.3 weeks [19]. This suggests that, after six months of prednisone therapy, the response rate is likely to be low. In fact, Cattran et al. reported that if a patient did not respond to steroids after six months, treatment beyond this duration was not beneficial [20]. Therefore, the persistence of NS after a longer course of therapy (four months of prednisone at a dosage of 1 mg/kg per day) can be used to define steroid resistance in nephrotic adults with FSGS. 

Upon comparing the laboratory results among adults and children, we observed a much worse pattern of results in the adult sample; thus, by looking at the medical approach used to treat FSGS in both age groups, we found that cyclosporin was prescribed to a lesser extent in adults than in children. Keeping in mind that the most common FSGS subtype in our adult patients was collapsing (a steroid-resistant form of FSGS), it was unusual that cyclosporin was not administered more frequently. Notably, the collapsing subtype was reported as the most aggressive form of FSGS. Additionally, it had the worst prognosis, was associated with severe NS, was steroid resistant, and had a rapid progression towards renal failure [5]. Likewise, another study also reported that the collapsing subtype of FSGS is steroid resistant and rapidly progresses to renal failure [21-22]. Another study found that the median renal survival among patients with the collapsing variant was significantly lower (13 months) than that observed in other subtypes (63 months), which may contribute to the former progressing towards ESRD [7]. We assume that the decline in the kidney function of adult patients with the collapsing subtype was because they did not receive cyclosporine as part of their treatment, as steroid-resistant FSGS requires dual therapy with glucocorticoids and cyclosporine. Based on a randomized clinical trial, the only drugs observed to be effective in increasing the rates of remission were cyclosporine combined with low-dose prednisolone [23]. Additionally, a study found that glucocorticoids and calcineurin inhibitors (cyclosporine) were successful in 50% of patients with FSGS [5]. Combined, glucocorticoids and calcineurin inhibitors may have a synergistic effect, as glucocorticoids have the benefit of regulating the actin cytoskeleton and proteins of the slit diaphragm, decreasing apoptosis and improving the podocyte differentiation marker following an injury [24]. Cyclosporine has a stabilizing effect on the acting cytoskeleton by preserving synaptopodin from breakdown [25]. Based on the findings from these studies, we suggest that collapsing subtypes respond poorly to glucocorticoid therapy and require additional immunosuppressive treatment such as cyclosporine, which may explain why our adult patients did not show as much improvement as pediatric patients, who were more exposed to cyclosporine. Therefore, we advise that the treatment approach to FSGS in adults should include cyclosporine combined with glucocorticoids.

The prevalence of FSGS is higher in males than in females

According to our findings, FSGS was significantly more prevalent in males than in females (55% vs 45%). In another study conducted in India, investigators identified only one female out of the 13 patients diagnosed with FSGS [21]. Our finding is in accordance with that of a previous Saudi Arabian study, which reported that only 28 of the 63 biopsies included were from females [13]. While the underlying cause of this sex discrepancy is unclear, it could be because hypertension is less prevalent in females. In fact, previous studies have reported that hypertension (mainly the malignant form) is strongly associated with the development of FSGS (especially the secondary type). For example, a study that included 10 cases of renovascular hypertension found FSGS in three out of the four kidney biopsies performed [20]. In another study, Kadiri et al. performed biopsies on 38 patients with malignant hypertension and found glomerulosclerosis in all of their samples [26]. Notably, some investigators have found that hypertension is more prominent in men [27-28]. Furthermore, a study that included patients from the United States, Australia, and Korea also reported that males were at higher risk for hypertension than females [27]. Finally, a prospective study assessing the impact of sex on primary glomerulosclerosis observed a male predominance, likely because the females in their study had less proteinuria and hypertension at the beginning of the study [28]. Therefore, based on our findings and those of the studies mentioned above, we hypothesize that men have a higher prevalence of FSGS than women because they are more prone to hypertension, which is a significant risk factor for the disease.

ESRD

FSGS is one of the most common primary glomerular diseases that leads to ESRD [29]. Over the course of three to six years, patients with nephrotic-range proteinuria appear to have the highest risk of developing ESRD [29]. In studies that included up to 20 years of follow-up data, the reported frequency of ESRD reached 78% [23]. As noticed in previous research, a prolonged follow-up period in patients with FSGS is necessary to reach the highest number of ESRD cases. This implies that the follow-up period is directly proportional to the cases of ESRD discovered [23]. The time between the onset of FSGS, which is indicated by proteinuria, and the onset of ESRD is typically three to 15 years (and probably longer) [7]. Unfortunately, in our study the follow-up period was short, which could explain why only 38% of our patients developed ESRD. This finding is consistent with prior research, which found that 50% of FSGS patients developed ESRD within eight years [24]. Data from another study showed that within six to eight years, 50% of patients with nephrotic-range proteinuria developed ESRD [25].

Limitations

Our research has some limitations that should be addressed in future studies. First, our sample size was not sufficiently large enough to fully assess the trends and outcomes of the disease; thus, a much larger sample size is needed to gain more insight and knowledge of FSGS. Furthermore, our study was limited to one center (KAUH), which represents only one hospital in Jeddah. Therefore, a multicenter study is necessary to confirm the generalizability of our observations on FSGS in Saudi Arabia.

## Conclusions

We found a high prevalence of collapsing and NOS subtypes in both adult and pediatric FSGS patients, with tip lesions being the least common. In addition, the most prevalent outcome was the persistence of NS, with low rates of remission. Future studies with a larger sample size are warranted to better understand this disease. This can be achieved by conducting a multicenter study, which would enable future researchers to generalize their results to a broader region. Additionally, future prospective studies will be helpful in confirming our proposed theories.
